# The importance of shell: Redating of the To’aga site (Ofu Island, Manu'a) and a revised chronology for the Lapita to Polynesian Plainware transition in Tonga and Sāmoa

**DOI:** 10.1371/journal.pone.0211990

**Published:** 2019-09-05

**Authors:** Fiona Petchey, Patrick V. Kirch

**Affiliations:** 1 Radiocarbon Dating Laboratory, Division of Health, Engineering, Computing and Science, University of Waikato, Hamilton, New Zealand; 2 ARC Centre of Excellence for Australian Biodiversity and Heritage, College of Arts, Society and Education, James Cook University, Cairns, QLD, Australia; 3 Department of Anthropology, University of California, Berkeley, United States of America; 4 Department of Anthropology, University of Hawai'i, Manoa, Hawai’i, United States of America; University at Buffalo - The State University of New York, UNITED STATES

## Abstract

Radiocarbon dating Pacific archaeological sites is fraught with difficulties. Often situated in coastal beach ridges or sand dunes, these sites exhibit horizontal and vertical disturbances, datable materials such as wood charcoal are typically highly degraded, may be derived from old trees or driftwood unless specifically identified to short-lived material, while bone collagen rarely survives in tropical conditions. Shell, therefore, is the most logical material for dating Pacific sites since it is resistant to alteration, can be sampled to ensure only the last few seasons of growth are represented and is often closely tied to human economic activities. However, shell radiocarbon (^14^C) dating has been plagued by interpretive problems largely due to our limited knowledge of the ^14^C cycle in nearshore marine and estuarine environments. Consequently, shell dates are typically ignored in regional chronometric evaluations and in recent years shell is often avoided for dating altogether. Recent advances in our understanding of the source of shell ^14^C as well as the development of the first South Pacific Gyre model of changing marine ^14^C over time, combined with Bayesian statistical modelling, now provide us with insight into the value of these shell radiocarbon dates. Here we present a revision of the age of the To’aga site on Ofu Island–an early occupation site associated with the initial Polynesian Plainware period in Sāmoa, the earliest use of which we date to between 2785 and 2607 cal BP (68% probability).

## Introduction

**Fig 1:**
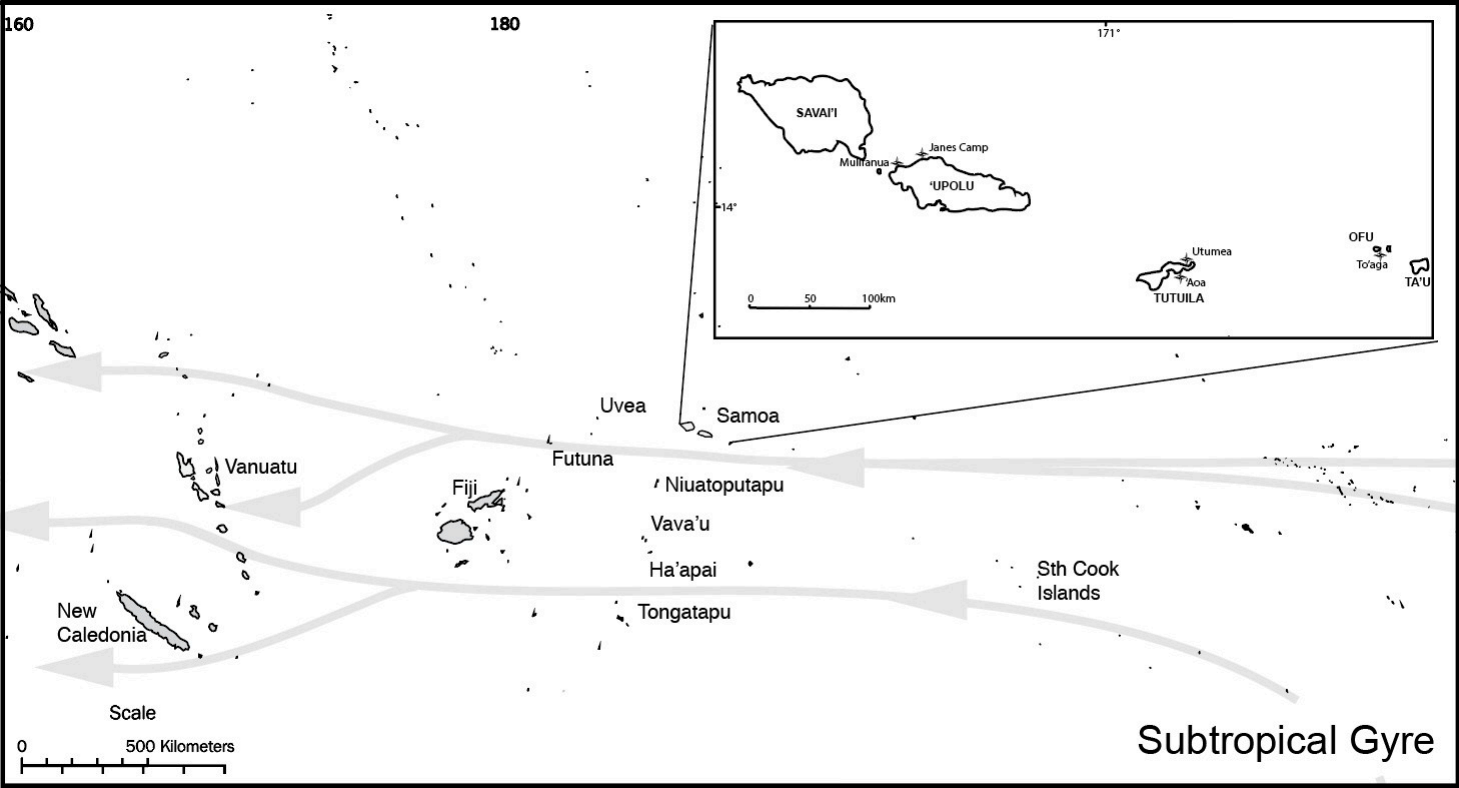
Map of the central subtropical gyre waters and associated island groups. Insert: Sāmoan Archipelago showing sites mentioned in text.

The first human presence in Remote Oceania can be mapped by the distribution of sites with dentate-stamped ceramic pottery that are found from southern Island Melanesia through to Western Polynesia and the northeastern coast of Australia (Fig 1). This Lapita culture is argued to have begun in the Bismarck Archipelago possibly as early as 3350 years ago, reaching its eastern-most extent in the Tongan and Sāmoan archipelagos around 2900–2750 BP. As Lapita people moved through the island groups they introduced a range of horticultural crops and domesticated animals to the region, providing the basis for subsistence systems that remain to this day. The timing of first landfall on each island group by these Lapita explorers has been the subject of a number of chronological evaluations (e.g., [1][2][3][4][5][6][7][8][9][10][11]) but many parts of this story remain controversial. Of note are debates over the timing of the gradual loss of dentate-stamped Lapita ceramics and their replacement with undecorated ceramics (termed Polynesian Plainware [PPW])—a significant change in material culture that is considered to mark the onset of Ancestral Polynesian society within a West Polynesian “homeland” [12]. Recently, Addison and Matisoo-Smith ([3], pg 7]) have drawn attention to a lack of quantitative data demonstrating continuity between Polynesian society and earlier Lapita groups, stressing that a greater level of complexity is likely.

There is no doubt that Lapita colonizers reached the island of 'Upolu in Western Sāmoa, where classic dentate-stamped Lapita ceramics of local manufacture were found at the submerged coastal site of Mulifanua (SU-17-1) [13][14]. The exact date and duration of this settlement remains somewhat problematic because the material was found following dredging during the construction of a ferry terminal [15]. Radiocarbon dates (here reported at 68% probability) obtained on shell and bone material collected from the dredging spoils produced a combined date of 2880–2750 cal BP [16]. Comparably early charcoal dates have also been obtained from the sites of ‘Aoa (AS-21-5) on Tutuila Island, and To‘aga (AS-13-1) on Ofu Island in the Manu'a group. Dates from these sites suggested that occupation either pre-dated, or was contemporaneous with Mulifanua [17][18] [19], but neither 'Aoa or To'aga produced ceramics with dentate stamped decoration. Instead, red- and orange-slipped thin, fine tempered plainware was recovered from the deepest units of both sites. These ceramics were considered to represent a distinct marker horizon between the earliest layers and those that contained thicker, coarse-tempered pottery that became dominant after 2400 cal BP ([20] pg 91).

Rieth and Hunt [21] evaluated all available ^14^C dates from the Sāmoan archipelago using a chronometric hygiene protocol. Chronometric hygiene is a classificatory procedure to assess the accuracy of temporal data using a set of minimum requirements that include material type, laboratory and context assessments. Similar methodologies have been widely applied throughout the Pacific (e.g., [22] [23] [24]). Rieth and Hunt’s [21] analysis challenged “the validity of the earliest dates from ‘Aoa and To‘aga” because of their low precision (standard errors in excess of ±100 years) ([21] pg 1921). This left only shell dates from To’aga for consideration, resulting in a ~300 year separation between Mulifanua and the earliest occupations at To‘aga (2500–2400 cal BP), Utumea (Tutuila Island; 2500–2100 cal BP; AS-22-44) and Jane’s Camp (‘Upolu; 2300–2000 cal BP; SU-18-1, SU-F1-1) ([21] pg 1916–1917). Addison and Morrison ([25] pg 363) subsequently concluded that the limitations of current radiocarbon technology and calibration methods, combined with the problems of stratification and mixing of sandy Pacific coastal sites, meant that further refinement of the absolute chronology for early Sāmoa was unlikely.

This post-2500 BP “re-colonization” date was questioned by Clark et al. [26] who presented at the Lapita conference in 2011 [27] new data from three PPW sites on the island of Ofu: Va’oto (AS-13-13), Coconut Grove (AS-13-37) and Ofu Village (AS-13-41). Combining these results in a single-phase Bayesian model, populated by a combination of short-lived *Cocos nucifera* endocarp charcoal and highly precise U/Th coral dates, Clark et al. ([26] pg 272) concluded that initial settlement of Ofu Island occurred between 2717 and 2663 cal BP (68% prob.). This overlapped with modelled dates for the end of Lapita from sites on Tongatapu, Ha’apai, and Vava’u (2703–2683 cal BP 68% prob.), where a chronological progression from Lapita to PPW sites had been identified [28], and suggested that the settlement of Ofu occurred soon after the loss of Lapita ceramics in Tonga. The question of whether the Lapita to PPW transition in Sāmoa represented two discrete settlement events, or a single transitional event was, however, unresolved.

Subsequent research in Sāmoa [29] [30] remains grounded in Rieth and Hunt’s [21] broad temporal framework, but advances in Tongan Lapita and PPW chronology [28] [29] and artifact provenance studies [30], which are significantly refined in comparison, have understandably contributed to a change in focus. In particular, this Tongan based research suggests contraction of established trade networks, which appear to have been completely severed with Sāmoa by ca. 2650–2600 BP ([31] pg, 235; though it is not clear how this date was calculated). Moreover, after several decades of archaeological investigation across the Sāmoan Archipelago, with no additional early sites, the general consensus is that the population immediately post-Lapita was severely diminished or absent [29] [32] [33]. Consequently, the development of Polynesian society within a joint Sāmoan/Tongan homeland has become less tenable, with the main support for this hypothesis largely confined to historical linguistics [34].

In their review of Sāmoan and Tongan prehistory, Burley and Addison ([31] pg 246) concluded that “Archaeology in Tonga and Sāmoa has reached the point where a secure chronology has been gained, and where informed questions can be asked.” We are less convinced of this. While new data using both ^14^C and new dating techniques (U/Th), combined with re-evaluation of existing data, provide great promise for resolving many chronological issues in the Pacific, the avoidance of key dating materials and the use of single-phase Bayesian evaluations with few dates, unconstrained by stratigraphy or other form of independent dating control, effectively leave us with an imprecise chronology that is smeared over many decades. Ultimately, we are still left with an inability to truly resolve important chronological questions: 1. When was the earliest occupation? 2. How fast did people spread? 3. Was settlement continuous? 4. From what direction did settlement spread?

In this paper, we address some of these issues by re-investigating the chronology of the To’aga site using a suite of new and precise shell and bone AMS ^14^C dates taken from key contexts. Based on these new ^14^C results, we then evaluate the placement of the To’aga site within the current chronological model for the Late Lapita/Polynesian transition throughout Sāmoa and Tonga.

## The To’aga excavation and radiocarbon dates

**Fig 2:**
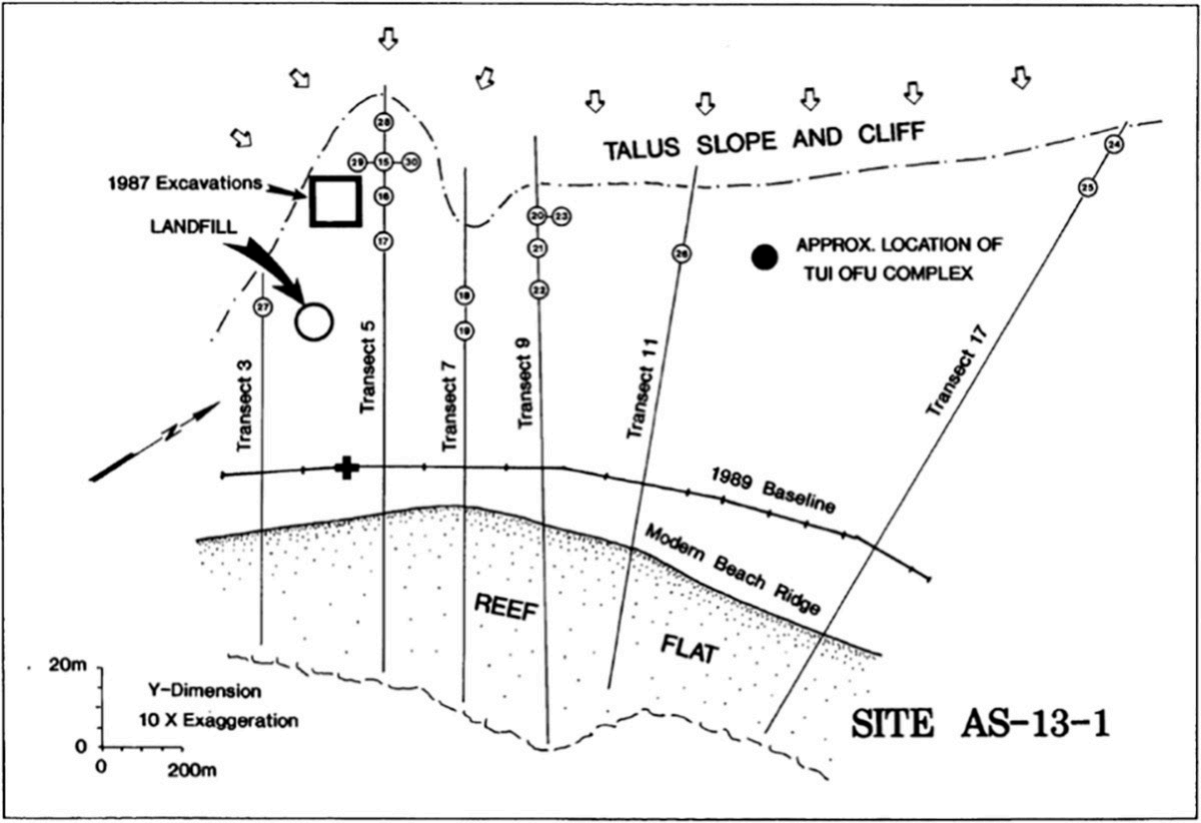
Map of the south eastern coast flat of Ofu Island showing location of excavations conducted in 1986, 1987, and 1989. **Numbered circles represent excavation units** (reproduced from [35] fig 5.8).

The To’aga site (AS-13-1), situated on the southern coast of Ofu Island, was first test excavated in 1986, with more extensive excavations being undertaken in 1987 and 1989 [19]. The site consists of stratified cultural deposits within a coastal beach terrace located on the southern side of Ofu Island. The terrace was archaeologically investigated primarily through the excavation of 1-m^2^ test pits arrayed along a series of six transects running perpendicular to the coast (Fig 2). These transects revealed buried cultural deposits, some of which contained PPW ceramics, shell fishhooks, ornaments, and other artefacts and associated faunal remains. During the 1987 field season an expanded trench was opened up to further sample the deeply buried, ceramic-bearing deposits, and is referred to below as the "Main Excavation". The original suite of radiocarbon dates from all three excavation seasons are presented in Kirch [20]. Calibrated ages at 68% probability are reproduced in this section as originally reported.

**Fig 3:**
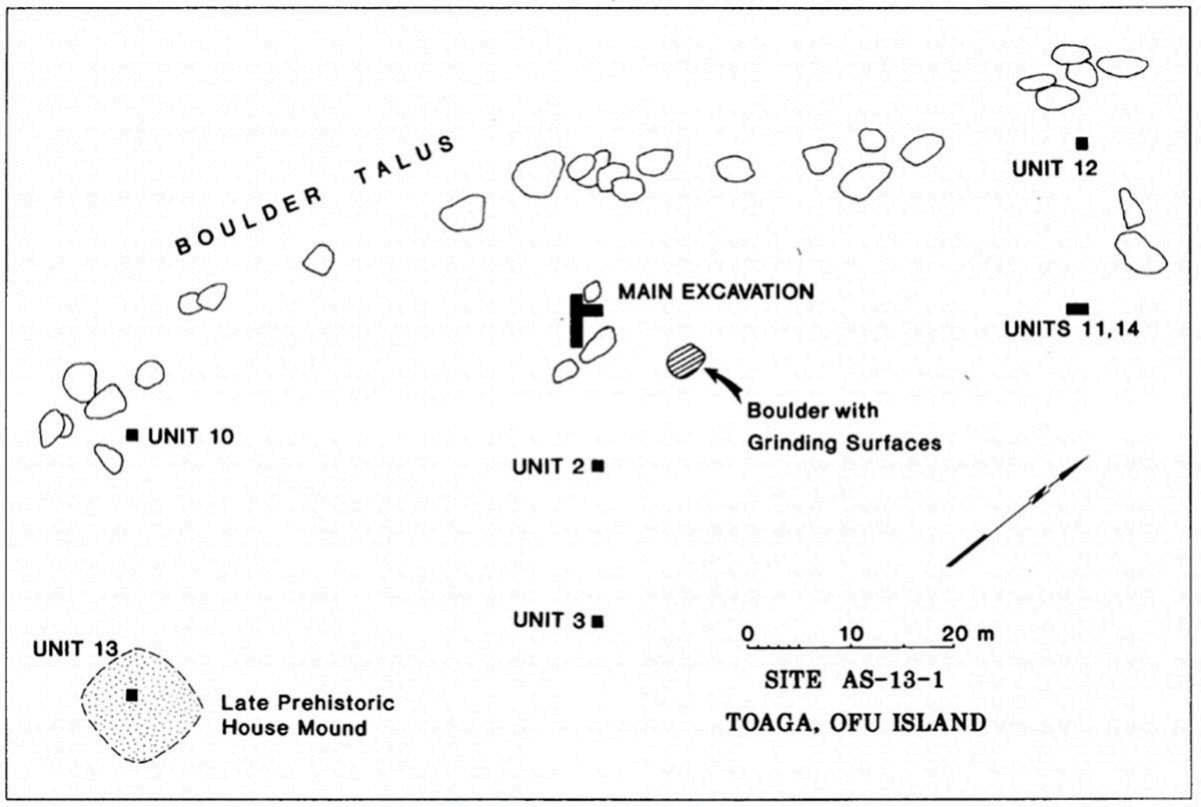
Map of the 1987 excavation area showing locations of Main Excavation and Unit 10 (reproduced from [35] fig 5.3).

For this renewed chronological evaluation, samples were sourced from excavated materials curated in the Oceanic Archaeology Laboratory at the University of California, Berkeley. These samples were specifically chosen to aid in defining the age of the oldest two main occupation deposits. They come from four locations; Unit 9 of the Main Excavation, Unit 10 located 45m to the southwest of the Main Excavation, Unit 28 located on Transect 5 to the east of the Main Excavation, and Unit 23 of Transect 9 also to the east of the Main Excavation (Figs 2 and 3). Radiocarbon dates and associated information are given in Table 1.

**Table 1:** New and extant radiocarbon dates from earliest layers at To’aga, Ofu Island.

Lab Code†	Provenance	Material	δ^13^C (‰)*	δ^18^O (‰)*	Conventional Radiocarbon Age (BP) plus error
**1987 MAIN EXCAVATION**					
Beta-25673#	Unit 1, Layer V	Shell: *Phalium* sp.	2.2	-	3620±80
Beta-25033	Unit 6, Layer IIA-1	Shell: *Turbo setosus*	2.3	-	2640±80
Beta-25034	Unit 6, Layer IIB	Shell: *Turbo setosus*	2.5	-	2570±80
Beta-25035#	Unit 6, Layer V	Shell: *Asaphis violascens* and *Lunella cincerea*	2.4	-	3820±70
$Wk-45468	Unit 9, Layer IIB	Shell: Echinoid spine	3.12	-0.28	2720±16
$Wk-45469	Unit 9, Layer IIB	Bone: *Rattus exulans* **	-17.8	-	2344±16
Beta-26464	Unit 10, Layer IIB	Charcoal: Unidentified	-27.8	-	2620±140
$Wk-45472	Unit 10, Layer IIB	Shell: Echinoid spine	2.68	-0.74	2761±16
$Wk-46708	Unit 10, Layer IIB	Shell: *Turbo setosus*	1.98	-1.62	2576±16
**TRANSECT 5**					
Beta-35601	Unit 28, Layer IIB (base)	Charcoal: Unidentified	-27.8	-	2900±110
$Wk-45473	Unit 28, Layer IIB (base)	Shell: Echinoid spine	2.25	0.34	2814±16
$Wk-46707	Unit 28, Layer IIB (base)	Shell: *Turbo setosus*	2.26	-1.47	2819±16
**TRANSECT 9**					
Beta-35602	Unit 23, Layer IIIA (Earth oven cut into B)	Charcoal: Unidentified	-26.9	-	2630±100
Beta-35603	Unit 23, Layer IIIB (base)	Charcoal: Unidentified	-28.4	-	2600±170
Beta-35604	Unit 23, Layer IIIB	Shell: *Tridacna maxima*	1.7	-	2770±80
$Wk-45470	Unit 23, Layer IIIB	Shell: Echinoid spine	2.69	-0.15	2809±17
$Wk-45471	Unit 23, Layer IIIB	Bone: *Rattus exulans*	-	-	2669±24
$Wk-47458	Unit 23, Layer IIIC	Shell: Echinoid spine	1.51	-1.56	2892±25
$Wk-47459	Unit 23, Layer IIIC	Shell: *Turbo* sp.	1.99	-2.08	2849±19

†Beta = Beta Analytic, Inc; Wk = Waikato Radiocarbon Dating Laboratory ($New data).

#Beta-25673 and Beta-25035 are not included in the chronometric model presented below because the results are considered unreliable (see text for detail).

* Environmental δ^13^C and δ^18^O values reported with Wk- dates were measured on solid shell using a using a cavity ring-down CO_2_ isotope analyser (CRDS) (Los Gatos Research model CCIA-46) at Waikato University using reference (NBS-19 and SDH synthetic CaCO3). Measured precision of ±0.35‰ for δ^13^C and ±0.40‰ for δ^18^O. δ^13^C and δ^18^O values are reported as ‰ V-PDB.

** δ^13^C (VPDB) values for rat dietary correction were measured by Isotope Ratio Mass Spectrometry (IRMS) at Iso-trace Research Department of Chemistry, University of Otago on a Carlo Erba NA 1500 elemental analyser (EA), coupled with either a Europa Scientific ‘20/20 Hydra’ or a Thermo Finnigan Delta Plus Advantage using reference (USGS-40, USGS-41) and control (EDTA-OAS and IAEA MP152) materials providing precision of ~±0.2‰ for δ^13^C.

### Main excavation

#### Original dates

The principal pottery-bearing layer in the Main Excavation is Layer IIB ([35] pg 51, fig 5.5). This is capped by the culturally sterile Layer IIA, with a thin zone on top (IIA-1) that contained scattered shell and coarse-tempered sherds interpreted to be eroded material that accumulated following the abandonment of occupation in this area. Two dates of *Turbo setosus* shell from Layer IIB gave equivalent results (Beta-25033; 2311–2094 cal BP, and Beta-25034; 2244–2007 cal BP). Towards the base of Layer IIC coral and volcanic rubble was interpreted as being the result of high energy deposition, most likely a storm event. Small numbers of thin, fine-tempered sherds were found in Layer IIC (a sandy, beach-ridge deposit) and in Layer III (massive colluvium derived from erosion up-slope); neither Layer IIC nor III were considered to represent in-situ occupations, and the sherds in them were interpreted as coming from the slope inland of the site. Layer IV was culturally sterile, and although two thin, fine-tempered sherds were recovered from Layer V these were also interpreted as having derived from an occupation locality further inland now buried by colluvium and not accessible through hand excavation ([35] pg 51, 56). A date of mixed shell taxa (Beta-25035; 3714–3549 cal BP) was obtained from lower Layer V, and a second date from nearby Unit 1 (Beta-25673; 3475–3326 cal BP) ([20] pg 91), but both results are considered to derive from secondary deposition and to be non-cultural. The dating of *Asaphis violascens* [Beta-25035], a deposit feeder is also a possible cause of this erroneous age [36]. The Unit 10 excavation exposed a stratigraphic sequence very similar to that of the Main Excavation, with Layer IIB considered to be identical to Layer IIB in the Main Excavation units (dated by wood charcoal sample Beta-26464; 2916–2403 cal BP) ([35] pg 53–54).

#### New dates

An additional four samples from the main cultural deposits (Layer IIB) in Units 9 and 10 were selected for dating; two dates of echinoid spines (Wk-45468 and Wk-45472), one of *Rattus exulans* bone (Wk-45469), and one of *Turbo setosus* shell (Wk-46708).

### Transect 5, Unit 28

#### Original dates

Transect 5 lay approximately 100m east of the Main Excavation ([35] fig 5.5). Unit 28 was placed at the base of a steep talus slope, and was specifically excavated to trace a deeply-buried cultural deposit exposed in Unit 15 (10m to the south of Unit 28) that contained a few sherds of thin, orange-slipped ceramics. Because the colluvial slope rises steeply along this inland edge of the coastal terrace, the excavation of Unit 28 required removal of 2.4m of colluvium and large boulders. Beneath the massive colluvium, the main cultural deposit (Layer II, with subcomponents IIB and IIC) contained ceramics and other artefacts and faunal material. Of 103 potsherds recovered from Layer IIC, 49 percent were of fine, thin-ware. A single charcoal ^14^C sample from the base of Layer II (Beta-35601; 3257–2879 cal BP) was considered to date in-situ cultural material. Because of the steeply rising colluvial slope inland of Unit 28, further excavations to the north were not possible, but it was thought likely that older deposits remained unexcavated in that direction ([35] pg 60, 67).

#### New dates

Two dates, from the same context as Beta-25601 (Transect 5, Unit 28), were obtained to confirm the age of Layer IIB; a date of an echinoid spine (Wk-45473) and a date of a *Turbo setosus* shell (Wk-46707).

### Transect 9, Units 20/23

#### Original dates

As with Unit 28, Units 20/23 were also located at the base of the talus slope, but about 400 m east of the Main Excavation. Layer IIIA was associated with a large earth oven feature dug down into Layer IIIB, containing large numbers of sea urchin spines (*Heterocentrotus mammillatus*), from which a charcoal ^14^C sample (Beta-35602; 2845–2612 cal BP) was originally dated. The underlying Layer IIIB was a thick, organic midden deposit that thinned into Layer IIIC ([35] pg 74, fig 5.22). Two charcoal dates (Beta-35603 and Beta-35602) from Unit 23, Layer III, returned ages of 2917–2382 cal BP and 2845–2612 cal BP respectively; significantly different from a shell date (Beta-35604; *Tridacna maxima*) from Layer IIIB (2444–2289 cal BP).

#### New dates

Four new dates were obtained from Unit 23; one of an echinoid spine (Wk-45470) and one of *Rattus exulans* bone (Wk-45471). Both came from the same context as the previous charcoal and *Tridacna* dates (i.e., Layer IIB). Two additional samples, an echinoid spine (Wk-47458) and a *Turbo* sp. shell (Wk-47459) from Layer IIIC, were dated to provide a maximum age for this part of the site.

### Comment on dates

The following evaluation of extant and new dates from To’aga does not follow the strict chronometric hygiene methodologies of previous researchers. Instead we favour a combination approach which applies both Bayesian statistical protocols—specifically outlier analysis [37]- with data transparency and critical evaluation of the dates in-line with current understanding of natural ^14^C variation [5].

#### Charcoal

Short-lived nut or twig charcoal samples with only 1 year of growth are considered to be one of the most reliable dating materials assuming there has been minimal stratigraphic displacement. It is also well-established that most unidentified “wood” charcoal determinations will date earlier than the event by an unknown amount [38], ranging from a few years up to several hundred years. Unfortunately, a large number of extant radiocarbon dates from Pacific contexts are of charcoal that has not been identified to short-lived materials, and removal of these unidentified charcoal dates from chronological evaluations would result in a dataset composed of relatively few dates. Pacific based research has indicated that, except in the highest precision analyses, minor inbuilt age typically goes unnoticed, and the impact is therefore overlooked [39] [40]. Bayesian outlier analysis specifically designed to account for this inbuilt age in charcoal is promising [5] [41], but typically requires additional constraints (e.g., dates on short-lived materials or stratigraphy) against which to anchor the model (see also [42] where the model code allows a small number of charcoal samples with inbuilt age to be younger than the context they represent, as would be the case with intrusive material). Once outliers have been correctly coded it has been demonstrated that a small dataset has a greater detrimental impact on chronological resolution than the potential of unidentified charcoals to skew the model to older ages [41].

### Marine shell

Marine shells that were gathered for food precisely date the timing of this activity. However, shell radiocarbon dates remain problematic because of uncertainties over what local ^14^C reservoir offset to apply (the ΔR value) to the modelled global marine ^14^C calibration curve. This value can vary because of upwelling, ocean circulation and connectivity of the animal’s habitat with the open ocean. This has resulted in many Pacific Island chronologies being dominated by wood and charcoal (e.g., [7] [43] [28] [44] [26]. See however, [5] [21] [45]). Petchey et al. [46] demonstrated little regional variation within the central Pacific Gyre zone, calculating an average ΔR value of 6±21 ^14^C years for modern shell collected from regions where currents are not interrupted by major island chains (e.g., Solomon Islands) or by contact between water bodies (e.g., Southland Front/Subtropical Front). Subsequent research has demonstrated that estuarine shells, in particular, may give apparently erroneous ^14^C results depending on genera and even species selected, as well as local near shore conditions, unless appropriate corrections are applied. Within the South Pacific Gyre region one major concern when dating molluscs is the presence of ancient limestone on many islands [47] which can be taken up by molluscs, either by directly living in those waters that have percolated through the limestone, or by algal grazing on the rock [48] [45]. Although *Turbo* and *Trochus* are potentially problematic in this respect ([49] [50] [36]), and sea urchins (echinoids) could similarly be affected because of their scavenging behaviour [51], the volcanic geology of Ofu Island minimalizes any problems of this nature [52].

**Fig 4:**
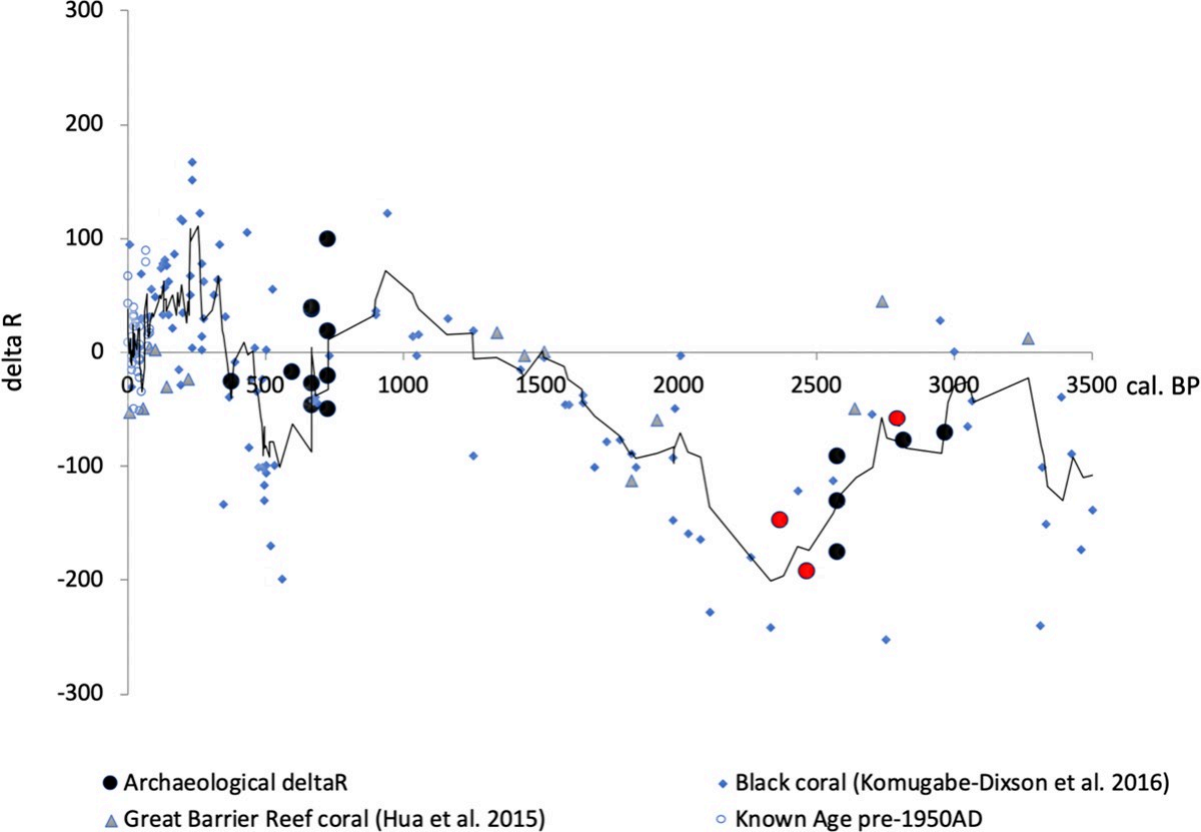
Change in delta R (ΔR) value across the Pacific over the last 3500 years. Red circles = U/Th coral samples from Va’oto and Coconut Grove (Ofu Island). Black trendline based on a 4-point moving average.

Recently, it has become apparent that ΔR values have not remained stable over the last 3000 years; shifts in marine ^14^C between 3000- and 1900-years ago, linked to changes in ocean circulation, have been documented in corals from the eastern coastline of Australia (Fig 4) [53] [54]. These ΔR changes have also been documented in archaeological shell specimens from the central South Pacific Gyre region [55]. ΔR values obtained from U/Th dated corals from Ofu Island, reported in Clark et al. [56], corroborate these observations. Consequently, modern reservoir correction values may not be applicable to archaeological material. The ΔR values available for the period between 3100 and 2600 BP have a pooled value of -48±82 ^14^C years. Between 2600 and 2250 BP the value drops to -160±48 ^14^C years (see S1 Table for ΔR values used to calculate these temporal average values). Unfortunately, this change occurs at a critical time for To’aga and the Lapita/PPW transition, and necessitates our testing both values.

#### Bone

Radiocarbon dates of *Rattus exulans* (Pacific rat) have a notoriety stemming from anomalously early results in several New Zealand archaeological contexts [57]. A range of theories have been put forward, including the small size of these bones and potential laboratory contamination at the time of dating [58] [59]. Twenty years later, there have been improvements in our understanding of dietary offsets and application of (dietary and reservoir) corrections to a range of animals that feed in both marine and terrestrial environments, as well as significant improvements in sample pretreatment and the abilities of AMS dating technology to date these tiny samples [60] [9] [61] [62] [63] [64]. The importance of rats as human commensals, as evidenced by their presence in many archaeological deposits including To’aga ([19] pg 200, table 13.3), means there is significant benefit from being able to date these animals directly [65]. They do however, require a dietary correction to account for marine and terrestrial ^14^C contributions. This can be calculated from δ^15^N and δ^13^C measured on the bone collagen and/or δ^13^C of bone carbonate [see S1 Text for methodology]. We are not aware of any rat specific ^14^C studies into dietary corrections for island environments, but evaluation of ^14^C dates of other omnivorous animals (i.e., humans, pigs and chickens) from Pacific contexts [9] [60] suggests a similar correction methodology is required.

#### Calibration

All radiocarbon dates were calibrated using OxCal v4.3.2 [66] with the Marine13 or Intcal13 curves [67]. We calibrated the shell results using either a ΔR of -48±82 ^14^C years or -160±48 ^14^C years and have produced three separate models depending on whether -48, -160 or a combination of both ΔR values has been used. We have used the Northern Hemisphere ^14^C calibration curve (Intcal13) due to the position of Ofu within the Sub-Tropical Convergence Zone (after [68]), though a mixture of the Southern and Northern Hemisphere calibration curves is likely to be more appropriate (cf. [69]), but the exact mix cannot currently be evaluated.

*Rattus exulans* bone dates require a dietary correction to the raw ^14^C data to obtain calibrated ages). A percent marine carbon (%MarineC, in this instance 28±10%) contribution to the diet was calculated for Wk-45469 which had a measured δ^13^C value of -17.8‰. The same correction, but with a ±20% uncertainty, was used for Wk-45471 because it was too small to also measure δ^13^C. Following the methodology outlined in Petchey et al. [70], both dates were calibrated with a corresponding mixture of the Intcal13 and Marine13. The radiocarbon determinations and stable isotope values can be found in Table 1.

#### Bayesian analysis

To determine the most probable chronology for To’aga, we conducted a Bayesian Sequence Analysis using OxCal 4.3.2 whereby radiocarbon ages are ordered on the basis of stratigraphic observations. In this model we have grouped the dates into two phases; Early (Transect 9, Unit 23 and Transect 5, Unit 28) and Main Excavation Layer (Units 6, 9 and 10) separated by a contiguous boundary. Within this “Early” phase, Unit 23 is further divided into three phases within a separate sequence that overlaps with the dates from Unit 28. No evidence of a hiatus in the deposition has been noted in the archaeology despite an apparent shift in activity foci [71] (Chronological Query language [CQL] code for the To’aga model is given in S2 Text).

**Fig 5:**
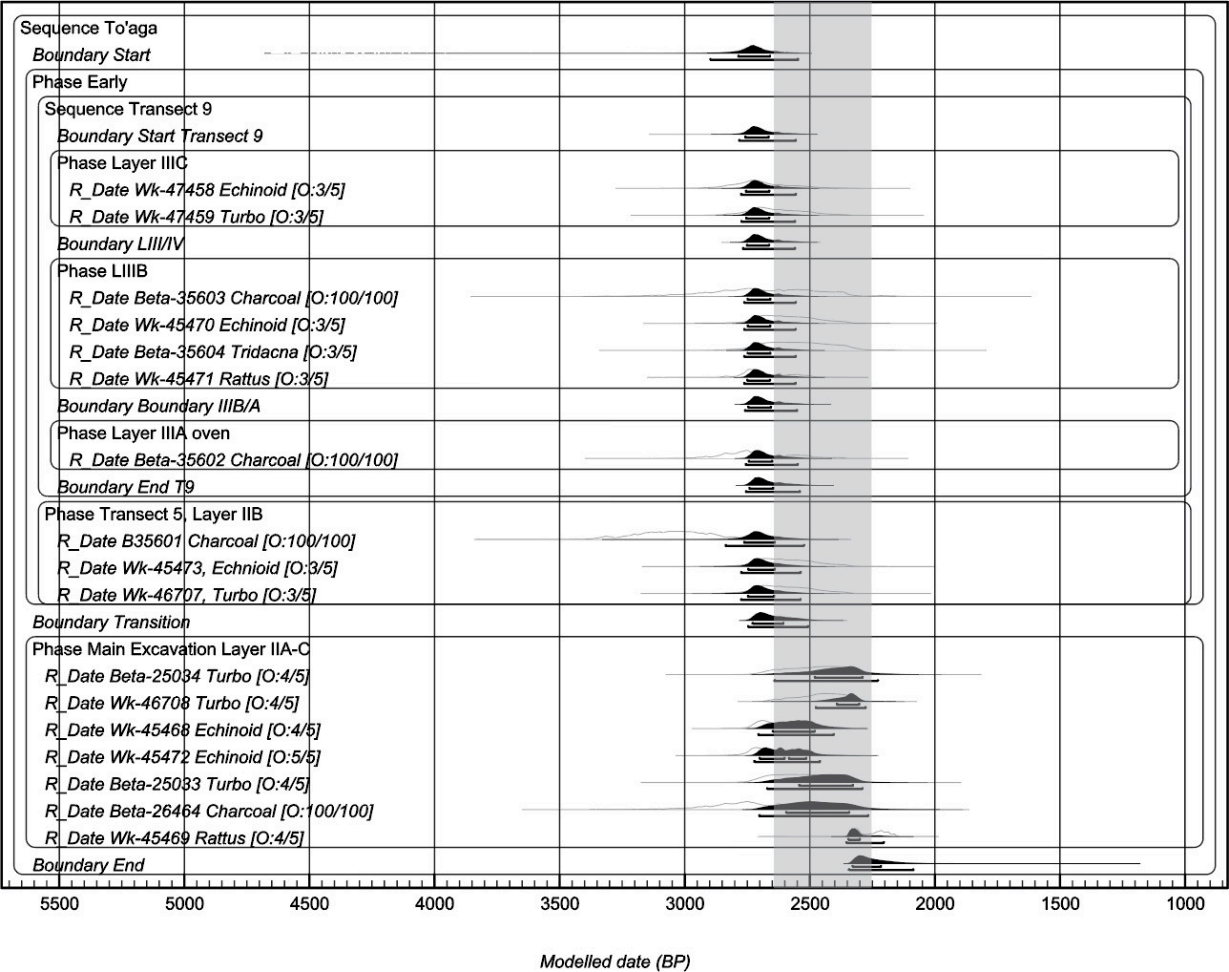
Bayesian sequence model for the To’aga site. This model uses a ΔR value of -160±48 ^14^C years for samples that date between 2600 and 2250 cal BP (i.e., “Main Excavation Layer IIA-C”) and -48±82 ^14^C years for earlier phases. The period between 2600 and 2250 cal BP is indicated by the grey bar. 68% and 95% error margins are indicated by bars under each age distribution. The notation [O:2/5] indicates a 2% posterior probability of being an outlier in the model.

To assess the likelihood of any one sample being an outlier, a General t-type Outlier Model is inset into the sequence [37]. This enables outliers to be either too young or too old and down-weights their influence in the model [38]. These dates are assigned a prior outlier probability of 0.05 and the scale of the offset is allowed to range anywhere between 10^0^ and 10^4^ years [“U(0,4)”]. The unidentified charcoal dates with possible inbuilt age are further assessed using an outlier correction for charcoal, as described by Bronk Ramsey [38]; (Exp (1,–10,0), U(0,3),‘t’), whereby the exponential distribution runs from -10 to 0 with a time-constant of 1, ensuring outliers can only be older. The shifts are then scaled by a common scaling factor that can lie anywhere between 10^0^ and 10^3^ years. The impact of outliers on the model can be assessed by the convergence values generated (S2, S3 and S4 Tables). These should be >95%. Lower values indicate many different incompatible solutions to the model at these points. By using outlier analysis all dates are independently assessed according to the model. In Fig 5, calibrated ages before the model parameters have been applied (“prior probability values”) are shown as unfilled outlines. Posterior probability values after the model has been applied are shown in black. Modelled calibrated dates are reported at 68% probability throughout the text. 95% probability values are given in Table 2 and in the supporting information.

**Table 2:** Radiocarbon Bayesian model results for To’aga depending on ΔR used.

	Model boundary ages (68% probability)	Model boundary ages (95% probability)
ΔR = -48±82	No outliers	
	Boundary Start: **2774–2568** cal BP	Boundary Start: **2964–2490** cal BP
	Boundary Transition: **2627–2439** cal BP	Boundary Transition: **2626–2384** cal BP
	Boundary End: **2301–2159** cal BP	Boundary End: **2330–2036** cal BP
ΔR = -160±48	No outliers	
	Boundary Start:**2809–2740** cal BP	Boundary Start: **2915–2722** cal BP
	Boundary Transition: **2754–2705** cal BP	Boundary Transition: **2772–2652** cal BP
	Boundary End: **2339–2257** cal BP	Boundary End: **2349–2129** cal BP
Both	No outliers	
	Boundary Start: **2785–2660** cal BP	Boundary Start: **2898–2547** cal BP
	Boundary Transition: **2729–2607** cal BP	Boundary Transition: **2745–2508** cal BP
	Boundary End: **2329–2216** cal BP	Boundary End: **2344–2086** cal BP

## Results

There are small differences in model results depending on whether a ΔR value of -48±82 or -160±48 ^14^C years, or a combination of ΔR values has been used (Table 2 and S2, S3 and S4 Tables). Convergence values generated by OxCal for all three models were uniformly high (>96.4) and therefore indicate that all three models are robust at the level of precision encountered and prior constraints applied. The use of the -48 ΔR value results in the ages hitting a plateau in the ^14^C levels–the “Radiocarbon Plateau” (ca. 2650–2350 cal BP). If the -160 value is used the calibrated dates are pushed back to before the plateau which results in an older age for the site. However, available evidence suggests this would be the incorrect ΔR to use at this time; use of the -160±48 ^14^C years ΔR, argued above to be required for the period 2600–2250 cal BP, results in a start boundary date for the site of 2809–2740 cal BP–an age which would negate the use of this ΔR value. The slightly later deposits in Layer IIB in the Main Excavation are, however, likely to date to after 2600 cal BP, in which case a ΔR of -160±48 ^14^C years can be argued to be appropriate for shell from these layers. However, the Main Excavation Layer IIA-C deposits almost certainly include a mixture of early and later material based on the ceramic evidence (i.e., the proportions of thin-ware versus thick-ware). This caveat aside, we favour a third model where both ΔR values are applied depending on an early or late designation (Fig 5 and S4 Table). Because the lower deposits are constrained in the multiphase sequence model by later deposits from the Main Excavation the influence of the Radiocarbon Plateau on the tail end of the distribution is minimalised regardless of the ΔR value used. This produces a date for the initial settlement of To’aga of between 2785 and 2607 cal BP at 68% probability (the latest possible end of this earliest phase of activity is represented by boundary “Transition” in Table 2 and Fig 5). Subsequent activities in the Main Excavation continued until 2216 cal BP. Ultimately, this model would benefit from more precise dates of materials of secure cultural association. It is also limited by our understanding of marine reservoir variation and that of the atmospheric ^14^C gradient between northern and southern hemispheres.

## Discussion

This dual ΔR multiphase sequence and outlier analysis provides the most secure age for the earliest deposits at To’aga available so far, and places first use of the site between 2785 and 2607 cal BP. This confirms the early age of the site, but how does the new boundary age compare to the chronology of Lapita and PPW in Sāmoa and Tonga? Moreover, is it possible to shed any further light on the key chronological questions for the region: when was the earliest occupation; how fast did people spread; was settlement continuous; and from what direction did settlement spread? In an attempt to answer these questions, and to highlight discrepancies in our current knowledge of regional chronology and radiocarbon methodology, the following regional comparison has been undertaken.

To ensure consistency across the datasets we have recalibrated the dates reported by Burley et al. [10] using the Intcal13 calibration curve. The original Tongan chronology presented by Burley et al. ([10] [44]) uses the Southern Hemisphere terrestrial calibration curve [72] while Clark et al. [56] uses the Northern Hemisphere curve (Intcal13). The average difference between the two curves up to 1000 BP is 41±14 ^14^C years. We added 16 new PPW dates reported by Burley et al. [44], and while we have duplicated the overlapping phase Bayesian analysis used in the 2015 study we have also added outlier analysis to the OxCal code; specifying either General t-type outlier for short-lived material or a Charcoal outlier in situations where the charcoal has not positively been identified to short-lived material (CQL code is given in S2 Text).

Similarly, we have rerun the single-phase model for Ofu Island (excluding dates from To’aga) using both short-lived and wood charcoal dates presented in Clark et al. [26], again with outlier analysis applied (CQL code is given in S2 Text). The Ofu PPW model originally reported by Clark et al. ([26] pg 271) consists of short-lived charcoal and U/Th dates of coral from the sites of Ofu Village, Coconut Grove and Va’oto. Their model was at risk of being biased by sampling and material choice. In particular, highly precise U/Th coral dates from Coconut Grove are not definitively associated with cultural activity. Although U/Th date 2014–19 may provide a *terminus post quem* for the formation of the earliest cultural layers (2014–19 came from the boundary of the lowest cultural layer and the paleo beach at Coconut Grove) the coral could have grown, and subsequently been deposited, prior to first site use ([26] pg 269). Consequently, in our revision of the Ofu PPW chronology we have applied a General t-type outlier to the U/Th dates to highlight that any measurement on this material dates the age of coral growth, not necessarily the age of the cultural modification. We have also applied the Charcoal outlier to all wood charcoal dates and the General t-type outlier to short-lived charcoal dates. This brings the total number of samples in the model to 23.

In the first model run (Model #1) of these 23 dates from Ofu Island the start boundary is dated to 2732–2519 cal BP (68% prob.), slightly later, but overlapping with the age range for initial occupation at To’aga (Table 3). However, U/Th date 2014–19 is highlighted as an outlier at 33% (i.e., has an impact on the model 67% of the time) and the start boundary has low a convergence of 66% (S5 Table). If we remove 2014–19 from the model the start of occupation begins 2570–2510 cal BP (68% prob.) (Model #2) and initial boundary convergence increases (98.7%) (S5 Table). However, within this single-phase model unconstrained by stratigraphy, the Ofu Village and Coconut Grove charcoal dates are now pulled into line with the highly precise U/Th dates from Va’oto—a site considered to be younger on artefactual evidence [26]. Our model results for To’aga indicate that human presence at this location began ca. 2785–2660 years ago, and continued until 2329–2216 cal BP. Therefore, Model #1 is not unreasonable, but on current evidence it is impossible to fully assess whether the age of first use of Coconut Grove (3 dates) and Ofu Village (2 dates) is comparable to To’aga (the earliest of these three sites–Coconut Grove–has only 3 dates of which one is U/Th sample 2014–19, the other two dates are of charcoal of very different ages). There is, however, little doubt that Ofu Island was, at the level of precision afforded by available ^14^C dates, continuously occupied at a time when Polynesian Plainware settlement in Tonga was well-established.

**Table 3:** Results from Bayesian single phase outlier analysis for Ofu Island (Coconut Grove, Ofu Village and Va’oto).

		68% prob.	95% prob.	Outliers
Model #1	Start	2732–2658 cal BP (58.4%)	2767–2639 cal BP (68.3%)	1 (2014–19); [O:33/5]
		2547–2519 cal BP (9.8%)	2614–2501 cal BP (27.1%)	
	End	2314–2241 cal BP	2326–2161 cal BP	
Model #2	Start	2570–2510 cal BP	2638–2498 cal BP	0
	End	2316–2267 cal BP	2330–2206 cal BP	

**Fig 6:**
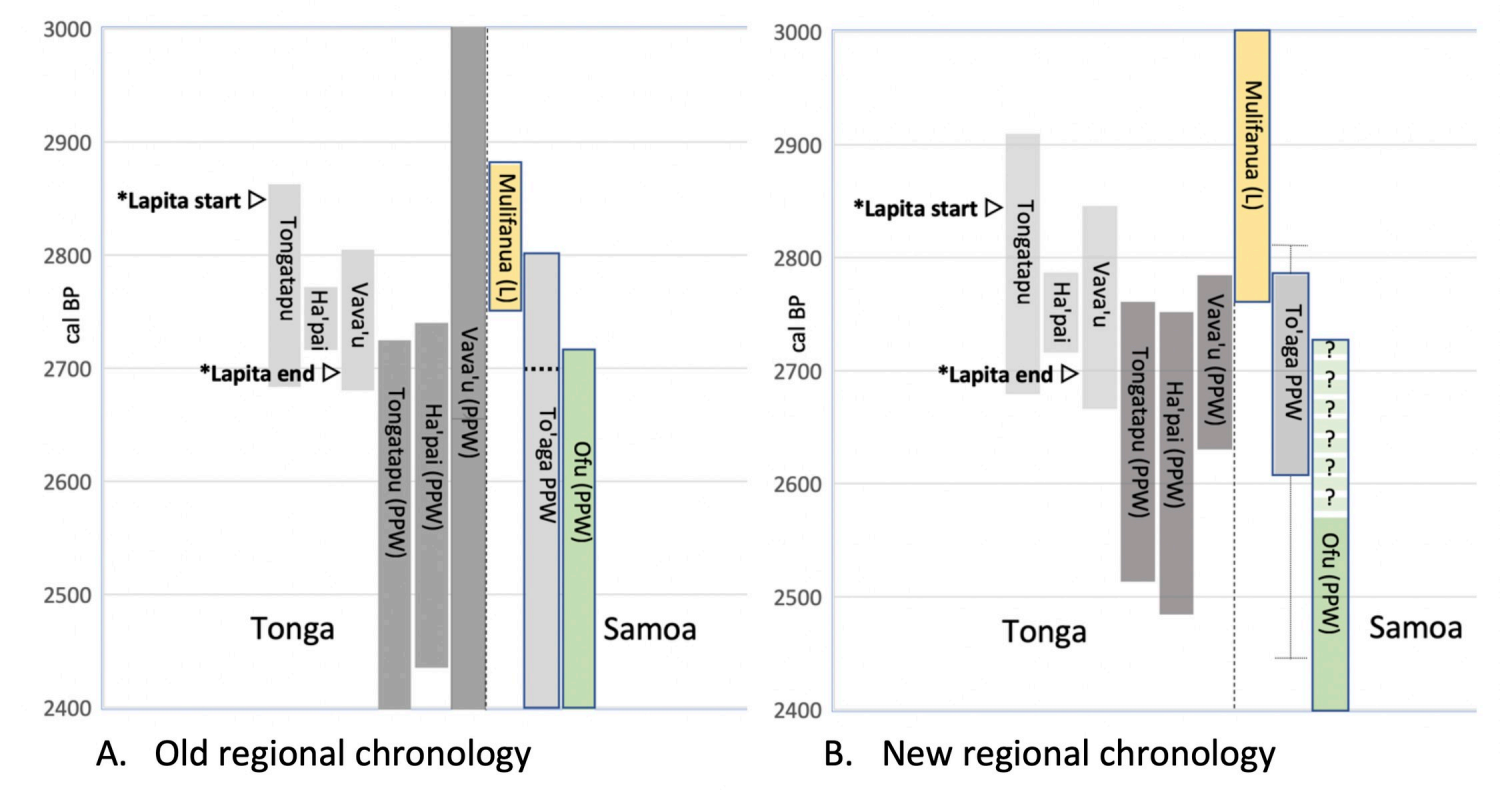
Regional chronology for Tonga and Sāmoa. **A.** Old regional chronology based on Clark et al. [26] for Sāmoan Polynesian Plainware sites (short-lived material only), Mulifanua [16], and Burley et al. [28] for Tongan Lapita and PPW sites. The range for To’aga is as reported by Kirch [20], pg 91). The approximated 95% age range for To’aga, as determined by [21] using shell dates, is 2700–2400 cal BP (upper estimate represented by dashed line on the To’aga bar). Lapita start and end arrows mark U/Th dates considered to represent the most secure age for Lapita start and end on Tongatapu and Vava’u respectively ([28] pg 10–11). **B.** New regional chronology using age-appropriate ΔR for To’aga, Mulifanua (NZA-5800 only), and PPW dates from Ofu [26] (excluding To’aga). Intcal13 has been used for all charcoal calibrations and dates from Burley et al. [44] have been incorporated into the PPW Tongan ranges. ? symbols represent the possible early (Model #1) age range for Ofu Island. The solid dark grey bar for To’aga represents the earliest phase of activity at the site only, with the whisker extensions representing the oldest and youngest end of the age range obtained for the earliest deposits depending on whether a ΔR of -48 or -160 is used. L = Lapita sites; PPW = Polynesian Plainware sites.

Fig 6 shows several important differences between the old regional chronology (6a) and proposed new chronology (6b). The slightly increased age range for the three Tongan island groups during the Lapita phase is because of the large number of unidentified charcoal dates down-weighted by the outlier analysis. The slight shift to older ages, most obvious in the Tongan Lapita phase, is a consequence of using the Intcal13 curve. The refinement to the PPW sequence is the result of the increased number of dates now available, and indicates the importance of larger numbers of dates when undertaking single-phase analyses of this type [41]. The start date for settlement of the Ofu Island, based on Coconut Grove, Ofu Village and Va’oto, is variable with two possible ages based on Model #1 and #2 outcomes outlined above, neither of which can be favoured on current published evidence.

The age for the Mulifanua Lapita site on ‘Upolu Island is now based on a single bone date (NZA-4780; 3062 ± 66 BP) rather than the more precise pooled result calculated by Petchey [16], and has shifted to a slightly older age range (3020–2760 cal BP, 68% probability) because of the use of the -48±82 ^14^C year ΔR value as opposed to a ΔR of 57±23 ^14^C years used previously [16]. Unfortunately, this date was measured over 20 years ago, and the available quality control information indicate that it would not pass bone assessment protocols now considered essential for an accurate ^14^C result (S1 Text).

### Directionality, continuity and speed

Burley et al. [28] argued for directionality with Lapita settlement moving from Tongatapu, where there was a short hiatus of ca. 70–90 years, before continued expansion through Ha'apai with near simultaneous movement into the Vava’u group; a maximum span of 158 years of Lapita settlement in Tonga. Our new multiphase model (Fig 6B) expands this to 243 years because of the additional uncertainty built into our outlier model. We cannot, however, detect evidence of a significant temporal change between settlements on Ha’pai and Vava’u, though Lapita settlements on Tongatapu do appear to be earlier. The argument that colonization occurred in a near-linear fashion, with Sāmoa settled later in the sequence ([28] pg 11), is also difficult to reconcile given ceramic evidence of connectivity between Fiji and Mulifanua [14] [73]. Given the similarity in age of Mulifanua to early Lapita sites on Tongatapu we note that contact with 'Upolu could have occurred relatively early in the sequence, predating later Lapita settlements in Ha’pai and Vava’u, thus reopening the possibility of Lapita settlement of Sāmoa via a northern corridor, through Futuna and ‘Uvea [74] [75], shortly after initial Lapita settlement on Tongatapu. This conclusion is tenuous, however, and necessitates further investigation of the antiquity of Mulifanua and these remote island outliers.

In the model presented by Burley et al. [28], the change to PPW occurred across the Tongan archipelago at a similar time. Our revised temporal model is not currently precise enough to confirm that the transition to PPW in Sāmoa occurred after Late Lapita settlements in Tonga, even though this seems logical based on the absence of dentate stamped ceramics at To’aga, but it does support the conclusion of Burley et al. [28] that change was very rapid and near-simultaneous. The refined age for To’aga, and the new calibrated age for the Lapita site at Mulifanua overlap. While the age for Mulifanua is far from ideal, the gap identified by Rieth et al. [76] and Addison and Morrison [25] is now significantly reduced and a failure of Lapita colonists to establish permanent and enduring settlements in Sāmoa seems less likely. Whether the initial settlement at To’aga occurred at the terminal end of Tongan Lapita, or during the subsequent PPW phase is uncertain because the earliest age we have for To’aga currently overlaps both. The continued use of To’aga from 2607 cal BP onwards (i.e., the Main Excavation/Transitional boundary date [Table 2]) overlaps activities elsewhere on Ofu Island supporting continuous settlement by this time. A continuum of human presence in Sāmoa has previously been postulated by Anderson and Clark ([77] pg 415) ostensibly because the distinctive Sāmoan plainware ceramics would have necessitated some length of time to develop.

Even though we can refine the chronology of movement through the islands using U/Th dating, as has been done for Tongan Lapita sites, this has not been possible so far for To’aga. The use of U/Th dates has also not been as successful for defining PPW, in part, because of limited numbers of suitable culturally modified corals from key archaeological sites. Even the corpus of ^14^C dates of short-lived materials from early sites is limited (see [21]). While it is difficult to give any recommendations as to the number of dates required to improve our findings further, Schmid et al. ([41] pg 67) has suggested in large-scale single-phase models ~280 ^14^C dates will produce results of the highest precision, with the caveat that the most accurate results are achieved where sampling density is uniformly distributed (our evaluation included 129 dates in a mix of single-phase models and stratigraphically controlled multiphase analysis). While many researchers question the usefulness of using ^14^C dating across the “Radiocarbon Plateau” (ca. 2650–2350 cal BP) there is structure present during this 300-year flat section of the calibration curve which could be utilised if larger numbers of precise (±20 years or better) dates of short-lived material were obtained from secure multiphase contexts.

Clearly, more work is required across this region and time period before robust hypotheses of population size, distribution and connectedness during Lapita and immediately post-Lapita time periods can be made. Ultimately, we must remember that all models are wrong, but some are useful [78]. Our new chronology for Sāmoa/Tonga provides the parameters on which to further refine our knowledge of the first settlement and subsequent cultural development across these archipelagoes.

## Conclusions

Our new dates and re-analysis of the site chronology indicates that the best estimation of the initial use of the To’aga site is between 2785 and 2607 cal BP. This confirms the antiquity of the site relative to other PPW sites on Ofu Island in Sāmoa, but because the site of Mulifanua is poorly dated we cannot confirm an overlap between To’aga and Sāmoan Lapita settlement. It is, however, apparent that settlement in Sāmoa occurred early and is likely to have continued in a near unbroken sequence. Our findings also suggest that the initial occupation at To'aga was contemporary with the terminal Lapita/PPW transition in Tonga.

Over the decades, radiocarbon dates and the interpretation of those dates have become more sophisticated, but as research themes develop and new dating technologies become integral to the debates, it has more than ever become necessary to refine the issues–both in archaeological research and radiocarbon methodology–that still plague the chronological interpretation. Chronometric hygiene methodologies initially provided the means to some clarity, but unfortunately removed a high proportion of dates from consideration. The limited number of early sites throughout the Pacific means we cannot afford to ignore the evidence already collected and where data are no longer considered of highest precision it is essential that extant excavations with curated samples are revisited. Bayesian methodologies now offer new opportunities to test these assumptions, as do refinements in our understanding of ^14^C variation in both the marine and terrestrial reservoirs.

## Supporting information

S1 Table ΔR values for shell/coral and references for the period between 3100 and 2650 BP and 2650 and 2250 BP. (DOCX)

S2 Table Result of Bayesian sequence model using ΔR of -48 ^14^C years. (XLSX)

S3 Table Result of Bayesian sequence model using ΔR of -160 ^14^C years. (XLSX)

S4 Table Result of Bayesian sequence model using ΔR of -160 and -48 ^14^C years. (XLSX)

S5 Table Ofu Island Bayesian analysis. (XLSX)

S1 Text Additional information for ^14^C and stable isotope samples. (DOCX)

S2 Text Bayesian OxCal models. (DOCX)
