# Supplementary material for: The importance of shell: Redating of the To’aga site (Ofu Island, Manu'a) and a revised chronology for the Lapita to Polynesian Plainware transition in Tonga and Sāmoa
Source: PLoS One. 2019 Sep 5;14(9):e0211990. doi: 10.1371/journal.pone.0211990 (PMC6728074; doi:10.1371/journal.pone.0211990)
Supplement: S1 Table — (DOCX) [file pone.0211990.s001.docx]

**S1 Table: ΔR values for shell/coral and references for the period between 3100 and 2650 BP and 2650 and 2250 BP.**

| **Location/ Sample identifier** | **Mean cal BP** | **ΔR*** | **Reference** |
| --- | --- | --- | --- |
| Between 2600 and 3100 cal BP | | | |
| †Heron Reef, Australia (OZO784 A-BO-H1-11) | 2638 | -49±26 | [1] |
| †St Helens Hill, Tasman Sea (BC1-13) | 2700 | -54±48 | [2] |
| †Morton Bay (OZM651, IW2) | 2734 | 46±102 | [1] |
| †St Helens Hill, Tasman Sea (SH9#11) | 2752 | -252±46 | [2] |
| †Coconut Grove (Ofu 2014-15) | 2796 | -60±17 | [3] |
| ^Bourewa, Fiji Phase 6, A1A/A1D | 2820 | -95±49 | [5] |
| St Helens Hill (SH10-B10) | 2948 | 28±24 | [2] |
| ^Naigani, Fiji, Sq A5, oven | 2980 | -72±80 | [4] |
| †St Helens Hill, Tasman Sea (SH10-ii) | 2997 | 0±38 | [2] |
| †St Helens Hill, Tasman Sea (SH-10_B9) | 3046 | -64±41 | [2] |
| Average ΔR: -48.07±10.24; χ^2^ _9:0.05_ = 33.82< 16.92; GSD = 82.28 | | | |
|  |  |  |  |
| Between 2250 and 2600 cal BP | | | |
| †St Helens Hill, Tasman Sea (SH9-1) | 2257 | -180±30 | [2] |
| †St Helens Hill, Tasman Sea (SH9#8) | 2329 | -242±34 | [2] |
| †Va'oto (Ofu 2014-22) | 2375 | -149±25 | [3] |
| †Cascade Plateau, Tasman Sea (BC1-11) | 2429 | -121±32 | [2] |
| †Va'oto (Ofu 2014-16) | 2470 | -194±28 | [3] |
| †Cascade Plateau, Tasman Sea (BC1-12) | 2558 | -112±30 | [2] |
| ^Talasiu, Tongatapu TAL 11 | 2590 | -92±66 | [5] |
| ^Talasiu, Tongatapu TAL 12 | 2590 | -132±63 | [5] |
| ^Talasiu, Tongatapu TAL 13 | 2590 | -176±63 | [5] |
| ^Talisiu Pinctada | 2590 | -100±72 | New data |
| Average ΔR: -159.66±11.28; χ^2^ _9:0.05_ = 13.99 < 16.92; GSD = 47.67 | | | |

† ΔR values based on U/Th, ^14^C pairs.

^ ΔR values based on ^14^C shell/short-lived charcoal pairs. Previously reported archaeological paired shell/charcoal ΔR values for Samoa (e.g., [7] [8]) are excluded from this calculation because the paired charcoals selected for dating were not identified to short-lived materials.

* All ΔR values have been recalculated using the online tool found at http://calib.org/deltar [6].

**Bibliography**

[1] Hua Q, Webb G, Zhao J, Nothdurft L, Lybolt M, Price G, et al. Large variations in the Holocene marine radiocarbon reservoir effect reflect ocean circulation and climatic changes. *Earth and Planetary Science Letters.* 2015;422: 33-44.

[2] Komugabe-Dixson A, Fallon S, Eggins S, Thresher R. Radiocarbon evidence for mid-late Holocene changes in southwest Pacific Ocean circulation. *Paleoceanography.* 2016;31: 971-985. doi:10.1002/2016PA002929.

[3] Clark J, Quintus S, Weisler M, St Pierre E, Nothdurft L, Feng Y, et al. Marine reservoir correction for American Samoa using U-series and AMS dated corals. *Radiocarbon.* 2016;58(4): doi:10.1017/RDC.2016.53.

[4] Irwin G, Worthy T, Best S, Hawkins S, Carpenter J, Matararaba S. Further investigations at the Naigani Lapita site (VL 21/5), Fiji: excavation, radiocarbon dating and palaeofaunal extinction. *Journal of Pacific Archaeology.* 2011;2: 66-78.

[5] Petchey F, Clark G. Tongatapu Hardwater: Investigation into the ^14^C marine reservoir offset in lagoon, reef and open ocean environments of a limestone island. *Quaternary Geochronology.* 2011;6: 539-549. doi:10.1016/j.quageo.2011.08.001.

[6] Reimer R, Reimer P. An online application for ΔR calculation. *Radiocarbon.* 2017;59(5): 1623-1627.

[7] Cleghorn P, Shapiro W. Archaeological data recovery report for the proposed Ta‘u road reconstruction, at Faga and Fitiuta, Ta‘u Island, Manu‘a, American Samoa. American Samoa Historic Preservtion Office; 2000.

[8] Kirch P. Radiocarbon chronology of the To’aga site. In: Kirch P, Hunt T, editors. *The To;aga site: Three millennia of Polynesian occupation in the Manu'a Islands, American Samoa*. Berkeley: Contributions of the University of California Archaeological Research Facility No. 51. Berkeley: University of California; 1993. pp. 85-91.
