# Supplementary material for: The importance of shell: Redating of the To’aga site (Ofu Island, Manu'a) and a revised chronology for the Lapita to Polynesian Plainware transition in Tonga and Sāmoa
Source: PLoS One. 2019 Sep 5;14(9):e0211990. doi: 10.1371/journal.pone.0211990 (PMC6728074; doi:10.1371/journal.pone.0211990)
Supplement: S1 Text — (DOCX) [file pone.0211990.s006.docx]

**S1 Text: Additional information for ^14^C and stable isotope samples.**

The materials selected for radiocarbon dating were curated in the Oceanic Archaeology Laboratory at the University of California, Berkeley, and hence under the control of co-author Kirch; no permits were required for the analyses described in this paper. Specimens were destroyed in the process of radiocarbon dating so are not accessible for further research. However, all other midden samples from the To'aga site have now been deposited in the Bernice P. Bishop Museum (Honolulu), and are thus available for future research.

Radiocarbon samples were prepared in the AMS facility at the Radiocarbon Dating Laboratory, Waikato University [1]. Two whole Turbo shells, Wk-47459 and Wk-46707, were sampled by removing a ~10 mm-long and ~4 mm-wide (35–45 mg) section parallel to the margin/lip. Wk-46708 was fragmentary and the sample was taken from the body of the shell. The echinoids were heavily fragmented and could not be identified to species. Echinoids grow rapidly and most have a life span of <20 years. They are also scavengers [2] and could ingest sediment of varying age. From each of the spines, we removed two outer rings for dating. These rings are likely to represent a lunar cycle [3]. The shells/echinoids were etched in 0.1M HCl at 80°C to remove ∼45% of the surface. The shells were tested for recrystallization by Feigl staining to ensure only aragonite was present in the shell. Echinoids are naturally calcitic and cannot be evaluated in this manner, but the visible condition of the spines under 10x magnification did not indicate secondary contamination.

For the *Rattus exulans* bone samples, gelatin was extracted and purified using (cleaned Centriprep, Ultracel YM-30 filters), and freeze-dried. A date on second “discard fraction” from ultrafilter was sampled to test age of possible contamination because the small size/low gelatin yield prevented routine bone quality assessment tests (e.g., C/N, %C and %N). This discard fraction gave a date of 2534±16 ^14^C years indicating <100yrs of possible error due to contamination. δ^15^N and δ^13^C measurements provide information on trophic level and ^14^C reservoir – both essential for date interpretation and calibration ([4][5]). The calculated %MarineC was calculated using linear regression between δ^13^C terrestrial (-20 ‰) and marine (-12‰) endpoints determined by evaluation of measurements of modern flora and fauna from the Pacific (Swift et al. 2017) and observed variation in archaeological rat isotopes [1]. An uncertainty of ±10 (Wk-45469) or ±20 (Wk-45471) was added to each calibration. Unfortunately, the sample was too small to measure δ^15^N and confirm a marine origin for the majority of the food protein. This leaves open the possibility that the measured δ^13^C could be due to the consumption of C_4_ plants which is usually identified by a partial marine δ^13^C value and a terrestrial δ^15^N value. This has been recorded in prehistoric populations from Fiji and New Caledonia [5] and in some rat populations from Mangareva [6]. However, most Polynesian-introduced economic plants are C_3_.

Mulifanua turtle bone (NZA-5800): Records held at GNS Science indicate that this sample was initially treated by demineralisation followed by gelatinisation, but failed to produce a sample of sufficient size for dating. The reported date of 3062 ± 66 BP was subsequently obtained on crude collagen (acid demineralised bone) (pers. comm. Margaret Norris, Rafter Radiocarbon Laboratory Manager, 9 Jan 2018).

# Bibliography

[1] Waikato Radiocarbon Dating Laboratory. Waikato Radiocarbon Dating Laboratory AMS Processing Technical Report. 2017. Available from: http://www.radiocarbondating.com/operating-procedures/ams-dating. [Accessed Jan 2019].

[2] Follo J, Fautin D. Echinoidea. Animal Diversity Web. 2001. Available from: http://animaldiversity.ummz.umich.edu/site/accounts/information/Echinoidea.html. [Accessed 16 January 2019].

[3] Weber J. Origin of concentric banding in the spines of the tropical Echinoid Heterocentrotus. *Pacific Science.* 1969;XXIII: 452-466.

[4] Carvalho A, Petchey F. Stable isotope evidence of Neolithic Palaeodiets in the coastal regions of southern Portugal. *Journal of Island and Coastal Archaeology.* 2013;8(3): 361-383.

[5] Petchey F, Spriggs M, Leach F, Seed M, Sand C, Pietrusewsky M, et al. Testing the human factor: Radiocarbon dating the first peoples of the South Pacific. *Journal of Archaeological Science.* 2011;38: 29-44.

[6] Swift J, Miller M, Kirch P. Stable isotope analysis of Pacific rat (*Rattus exulans*) from archaeological sites in Mangareva (French Polynesia): The use of commensal species for understanding human activity and ecosystem change. *Environmental Archaeology.* 2007; 22(3): 283-297. doi: 10.1080/14614103.2016.1216933.
