# Supplementary material for: The importance of shell: Redating of the To’aga site (Ofu Island, Manu'a) and a revised chronology for the Lapita to Polynesian Plainware transition in Tonga and Sāmoa
Source: PLoS One. 2019 Sep 5;14(9):e0211990. doi: 10.1371/journal.pone.0211990 (PMC6728074; doi:10.1371/journal.pone.0211990)
Supplement: S2 Text — (DOCX) [file pone.0211990.s007.docx]

**S2 Text: Bayesian OxCal models**

**CODE for Sequence model for To’aga (Nth Hemisphere) using Both ΔR values**

Plot()

{

Outlier_Model("General",T(5),U(0,4),"t");

Outlier_Model("Charcoal",Exp(1,-10,0),U(0,3),"t");

Sequence("To'aga")

{

Boundary("Start");

Phase("Early")

{

Sequence("Transect 9")

{

Boundary("Start Transect 9");

Phase("Layer IIIC")

{

Curve("Marine13","Marine13.14c");

Delta_R("LocalMarine",-48,82);

R_Date("Wk-47458 Echinoid", 2892, 25)

{

Outlier("General", 0.05);

};

R_Date("Wk-47459 Turbo", 2849, 19)

{

Outlier("General", 0.05);

};

};

Boundary("LIII/IV");

Phase("LIIIB")

{

Curve("IntCal13","IntCal13.14c");

R_Date("Beta-35603 Charcoal", 2600, 170)

{

Outlier("Charcoal", 1);

};

Curve("Marine13","Marine13.14c");

Delta_R("LocalMarine",-48,82);

R_Date("Wk-45470 Echinoid", 2809, 17)

{

Outlier("General", 0.05);

};

R_Date("Beta-35604 Tridacna", 2770, 80)

{

Outlier("General", 0.05);

};

Mix_Curve("Mixed","IntCal13","LocalMarine",28,20);

R_Date("Wk-45471 Rattus", 2669, 24)

{

Outlier("General", 0.05);

};

};

Boundary("Boundary IIIB/A");

Phase("Layer IIIA oven")

{

Curve("IntCal13","IntCal13.14c");

R_Date("Beta-35602 Charcoal", 2630, 100)

{

Outlier("Charcoal", 1);

};

};

Boundary("End Transect 9");

};

Phase("Transect 5, Layer IIB")

{

Curve("IntCal13","IntCal13.14c");

R_Date("B35601 Charcoal", 2900, 110)

{

Outlier("Charcoal", 1);

};

Curve("Marine13","Marine13.14c");

Delta_R("LocalMarine",-48,82);

R_Date("Wk-45473, Echnioid", 2814, 16)

{

Outlier("General", 0.05);

};

R_Date("Wk-46707, Turbo", 2819, 16)

{

Outlier("General", 0.05);

};

};

};

Boundary("Transition");

Phase("Main Excavation Layer IIA-C")

{

Curve("Marine13","Marine13.14c");

Delta_R("LocalMarine",-160,48);

R_Date("Beta-25034 Turbo", 2570, 80)

{

Outlier("General", 0.05);

};

R_Date("Wk-46708 Turbo", 2576, 16)

{

Outlier("General", 0.05);

};

R_Date("Wk-45468 Echinoid", 2720, 16)

{

Outlier("General", 0.05);

};

R_Date("Wk-45472 Echinoid", 2761, 16)

{

Outlier("General", 0.05);

};

R_Date("Beta-25033 Turbo", 2640, 80)

{

Outlier("General", 0.05);

};

Curve("IntCal13","IntCal13.14c");

R_Date("Beta-26464 Charcoal", 2620, 140)

{

Outlier("Charcoal", 1);

};

Mix_Curve("Mixed","IntCal13","LocalMarine",28,10);

R_Date("Wk-45469 Rattus", 2344, 16)

{

Outlier("General", 0.05);

};

};

Boundary("End");

};

};

**CODE for Tongan Lapita Single Phase model (Nth Hemisphere): n= 44**

Plot()

{

Outlier_Model("General",T(5),U(0,4),"t");

Outlier_Model("Charcoal",Exp(1,-10,0),U(0,3),"t");

Phase("Lapita")

{

Sequence(Tongatapu)

{

Boundary("Start 1");

Phase("Tongatapu")

{

Curve("IntCal13","IntCal13.14c");

C_Date("Nukukeka/UTh 11-36", -888, 4)

{

Outlier("General", 0.05);

};

R_Date("Nukuleka/Wk-23710 SL char", 2811, 35)

{

Outlier("General", 0.05);

};

R_Date("Nukuleka/Wk-23708 char", 2836, 32)

{

Outlier("Charcoal", 1);

};

R_Date("Nukuleka/CAMS-59624 char", 2790, 50)

{

Outlier("Charcoal", 1);

};

R_Date("Ha'ateiho/CAMS-59623 char", 2730, 50)

{

Outlier("Charcoal", 1);

};

C_Date("Ha'ateiho/UTh 12-10", -849, 7)

{

Outlier("General", 0.05);

};

C_Date("Nukuleka/UTh 11-33", -848, 4)

{

Outlier("General", 0.05);

};

R_Date("Nukuleka/Wk-23707 char", 2696, 32)

{

Outlier("Charcoal", 1);

};

R_Date("Ha'ateiho/CAMS-59622 char", 2670, 40)

{

Outlier("Charcoal", 1);

};

C_Date("Nukuleka/UTh 11-24", -788, 7)

{

Outlier("General", 0.05);

};

C_Date("Nukuleka/UTh 11-29", -780, 4)

{

Outlier("General", 0.05);

};

C_Date("Nukuleka/UTh 11-23", -776, 4)

{

Outlier("General", 0.05);

};

C_Date("Nukuleka/UTh 11-22", -774, 4)

{

Outlier("General", 0.05);

};

R_Date("Ha'ateiho/CAMS-59621 char", 2540, 40)

{

Outlier("Charcoal", 1);

};

C_Date("Nukuleka/UTh 11-25", -754, 3)

{

Outlier("General", 0.05);

};

R_Date("Nukuleka/Wk-23709 char", 2536, 32)

{

Outlier("Charcoal", 1);

};

C_Date("Nukuleka/UTh 11-32", -752, 4)

{

Outlier("General", 0.05);

};

};

Boundary("End 1");

};

Sequence("Ha'apai")

{

Boundary("Start 2");

Phase("Ha'apai")

{

Curve("IntCal13","IntCal13.14c");

R_Date("Tongoleleka/Beta-134590 bone", 2730, 40)

{

Outlier("General", 0.05);

};

R_Date("Tongoleleka/CAMS-34561 char", 2720, 60)

{

Outlier("Charcoal", 1);

};

R_Date("Tongoleleka/Beta-134591 bone", 2700, 40)

{

Outlier("General", 0.05);

};

R_Date("Tongoleleka/CAMS-41514 char", 2690, 50)

{

Outlier("Charcoal", 1);

};

R_Date("Vaipuna/CAMS-41526 char", 2690, 50)

{

Outlier("Charcoal", 1);

};

R_Date("Tongoleleka/Beta-134592 bone", 2680, 50)

{

Outlier("General", 0.05);

};

R_Date("Tongoleleka/Beta-134589 bone", 2660, 40)

{

Outlier("General", 0.05);

};

R_Date("Tongoleleka/Beta-134588 bone", 2630, 40)

{

Outlier("General", 0.05);

};

R_Date("Pukotala/CAMS-41516 SL char", 2640, 60)

{

Outlier("General", 0.05);

};

R_Date("Mele Havea/CAMS-41520 SL char", 2640, 50)

{

Outlier("General", 0.05);

};

R_Date("Pukotala/CAMS-7147 SL char", 2630, 60)

{

Outlier("General", 0.05);

};

R_Date("Mele/CAMS-41522 char", 2620, 50)

{

Outlier("Charcoal", 1);

};

R_Date("Vaipuna/CAMS-41531 char", 2620, 50)

{

Outlier("Charcoal", 1);

};

R_Date("Faleloa/CAMS-41530 char", 2600, 50)

{

Outlier("Charcoal", 1);

};

C_Date("Vaipuna/UTh 12-40", -813, 5)

{

Outlier("General", 0.05);

};

R_Date("Faleloa/CAMS-8074 SL char", 2560, 60)

{

Outlier("General", 0.05);

};

R_Date("Faleloa/CAMS-7146 SL char", 2560, 60)

{

Outlier("General", 0.05);

};

C_Date("Tongoleleka/UTh 12-38", -790.5, 7.5)

{

Outlier("General", 0.05);

};

R_Date("Tongoleleka/CAMS-41530 char", 2560, 50)

{

Outlier("Charcoal", 1);

};

C_Date("Tongoleleka/UTh 12-39", -775, 5)

{

Outlier("General", 0.05);

};

C_Date("Faleloa/UTh 12-37", -777, 7)

{

Outlier("General", 0.05);

};

};

Boundary("End 2");

};

Sequence("Vava'u")

{

Boundary("Start 3");

Phase("Vava'u")

{

Curve("IntCal13","IntCal13.14c");

R_Date("Vuna/CAMS-111662 char", 2715, 35)

{

Outlier("Charcoal", 1);

};

R_Date("Otea/CAMS-119701 char", 2705, 35)

{

Outlier("Charcoal", 1);

};

R_Date("Falevai/CAMS-119696 char", 2685, 35)

{

Outlier("Charcoal", 1);

};

R_Date("Vuna/CAMS-111659 char", 2650, 35)

{

Outlier("Charcoal", 1);

};

R_Date("Ofu/CAMS-119696 SL char", 2625, 35)

{

Outlier("General", 0.05);

};

C_Date("Ofu/UTh 12-36", -753, 5)

{

Outlier("General", 0.05);

};

};

Boundary("End 3");

};

Span("Lenth of Lapita");

};

};

**CODE for Tongan PPW Single Phase model (Nth Hemisphere): n= 45**

Plot()

{

Outlier_Model("General",T(5),U(0,4),"t");

Outlier_Model("Charcoal",Exp(1,-10,0),U(0,3),"t");

Sequence("PPW")

{

Boundary("Start 1");

Phase("Tongatapu PPW")

{

R_Date("Ha'ateiho/UOC-3859 SL char ", 2583, 22)

{

Outlier("General", 0.05);

};

R_Date("Talasiu /Wk-23002 char", 2562, 30)

{

Outlier("Charcoal", 1);

};

R_Date("Talasiu /Wk-33572 SL char", 2553, 25)

{

Outlier("General", 0.05);

};

R_Date("Ha'ateiho/UOC-3860 char", 2542, 25)

{

Outlier("Charcoal", 1);

};

R_Date("Talasiu/Wk-28235 SL char", 2510, 31)

{

Outlier("General", 0.05);

};

R_Date("Talasiu/Wk-33574 SL char", 2504, 25)

{

Outlier("General", 0.05);

};

R_Date("Ha'ateiho/UOC-3861 char", 2499, 22)

{

Outlier("Charcoal", 1);

};

R_Date("Ha'ateiho/UOC-3862 char", 2493, 25)

{

Outlier("Charcoal", 1);

};

R_Date("Talasiu/Wk-28234 SL char", 2473, 31)

{

Outlier("General", 0.05);

};

R_Date("Moisa/SANU-54629 SL char", 2461, 32)

{

Outlier("General", 0.05);

};

R_Date("Talasiu/Wk-22876 char", 2452, 30)

{

Outlier("Charcoal", 1);

};

R_Date("Talasiu/Wk-33573 SL char", 2448, 25)

{

Outlier("General", 0.05);

};

R_Date("Fakala'a/SANU-54628 SL char", 2439, 38)

{

Outlier("General", 0.05);

};

R_Date("Tufumahina/NZ-636 char", 2380, 51)

{

Outlier("Charcoal", 1);

};

R_Date("Tongolekea/CAMS-34559 char", 2600, 60){

Outlier("Charcoal", 1);

};

R_Date("Holopeka/ CAMS-12919 char", 2439, 38)

{

Outlier("Charcoal", 1);

};

R_Date("Vaipuna/CAMS-41523 char", 2580, 50)

{

Outlier("Charcoal", 1);

};

R_Date("Vaipuna/CAMS-41525 char", 2560, 80)

{

Outlier("Charcoal", 1);

};

R_Date("Faleloa/CAMS-17146 char", 2560, 60)

{

Outlier("Charcoal", 1);

};

R_Date("Tongoleleka/ CAMS-34560 char", 2560, 50)

{

Outlier("Charcoal", 1);

};

R_Date("Pukotala/CAMS-41515 char", 2560, 50)

{

Outlier("Charcoal", 1);

};

R_Date("Faleloa/ CAMS-41529 char", 2550, 50)

{

Outlier("Charcoal", 1);

};

R_Date("Faleloa/CAMS-41529 SL char", 2550, 50)

{

Outlier("General", 0.05);

};

R_Date("Tongoleleka /UOC-3873 char", 2550, 23)

{

Outlier("Charcoal", 1);

};

R_Date("Holopeka/CAMS-41527 SL char", 2540, 50)

{

Outlier("General", 0.05);

};

R_Date("Pukotala/CAMS-41517 char", 2540, 50)

{

Outlier("Charcoal", 1);

};

R_Date("Tongoleleka/ UOC-3872 char", 2540, 24)

{

Outlier("Charcoal", 1);

};

R_Date("Holopeka/CAMS-41528 char", 2510, 50)

{

Outlier("Charcoal", 1);

};

R_Date("Mele/CAMS-41521 char", 2510, 50)

{

Outlier("Charcoal", 1);

};

R_Date("Mele/UOC-3868 SL char", 2505, 22)

{

Outlier("General", 0.05);

};

R_Date("Mele /UOC-3866 char", 2493, 22)

{

Outlier("Charcoal", 1);

};

R_Date("Mele /UOC-3869 SL char", 2491, 22)

{

Outlier("General", 0.05);

};

R_Date("Tongoleleka/CAMS-41512 SL char", 2490, 51)

{

Outlier("General", 0.05);

};

R_Date("Mele Havea/CAMS-41519 SL char", 2490, 50)

{

Outlier("General", 0.05);

};

R_Date("Mele/ UOC-3867 char", 2478, 22)

{

Outlier("Charcoal", 1);

};

R_Date("Tongoleleka/ UOC-3874 char", 2460, 22)

{

Outlier("Charcoal", 1);

};

R_Date("Tongoleleka/ CAMS 34558 char", 2450, 40)

{

Outlier("Charcoal", 1);

};

R_Date("Tongoleleka/CAMS-41513 SL char", 2430, 50)

{

Outlier("General", 0.05);

};

R_Date("Tongoleleka /Beta-14171 char", 2330, 60)

{

Outlier("Charcoal", 1);

};

R_Date("Falevai/CAMS-119695 char", 2645, 35)

{

Outlier("Charcoal", 1);

};

R_Date("Otea/ UOC-3865 char", 2572, 26)

{

Outlier("Charcoal", 1);

};

R_Date("Falevai/ UOC-3871 char", 2561, 25)

{

Outlier("Charcoal", 1);

};

R_Date("Otea/ UOC-3863 char", 2529, 29)

{

Outlier("Charcoal", 1);

};

R_Date("Falevai /UOC-3870 char", 2483, 22)

{

Outlier("Charcoal", 1);

};

R_Date("Falevai/CAMS-119694 char", 2500, 35)

{

Outlier("Charcoal", 1);

};

};

Boundary("End 1");

};

};

**CODE for Ofu PPW model #1 (Nth Hemisphere): n= 23**

Plot()

{

Outlier_Model("General",T(5),U(0,4),"t");

Outlier_Model("Charcoal",Exp(1,-10,0),U(0,3),"t");

Sequence("Ofu Samoa PPW")

{

Boundary("Start 1");

Phase("Ofu PPW")

{

R_Date("Va'oto/Beta-249325 char", 2200, 40)

{

Outlier("Charcoal", 1);

};

R_Date("Va'oto/Beta-128705 char", 2230, 40)

{

Outlier("Charcoal", 1);

};

R_Date("Va'oto/Beta-297826 char", 2280, 40)

{

Outlier("Charcoal", 1);

};

R_Date("Va'oto/Beta-366730 SL char", 2350, 30)

{

Outlier("General", 0.05);

};

R_Date("Va'oto/Beta-366729 SL char", 2350, 30)

{

Outlier("General", 0.05);

};

R_Date("Va'oto/Beta-262551 char", 2320, 50)

{

Outlier("Charcoal", 1);

};

R_Date("Va'oto/Beta-120417 char", 2370, 50)

{

Outlier("Charcoal", 1);

};

R_Date("Va'oto/Beta-249326 char", 2430, 40)

{

Outlier("Charcoal", 1);

};

R_Date("Va'oto/Beta-297824 char", 2520, 30)

{

Outlier("Charcoal", 1);

};

R_Date("Va'oto/Beta-249327 char", 2520, 40)

{

Outlier("Charcoal", 1);

};

R_Date("Va'oto/Beta-128706 char", 2460, 40)

{

Outlier("Charcoal", 1);

};

R_Date("Coconut Grove/Beta-308978 char", 2370, 30)

{

Outlier("Charcoal", 1);

};

R_Date("Coconut Grove/Beta-307473 SL char", 2470, 30)

{

Outlier("General", 0.05);

};

R_Date("Ofu/Beta-354137 SL char", 2490, 30)

{

Outlier("General", 0.05);

};

R_Date("Ofu/Beta-383081 SL char", 2490, 30)

{

Outlier("General", 0.05);

};

C_Date("Va'oto/UTh -16", -520, 8)

{

Outlier("General", 0.05);

};

C_Date("Va'oto/UTh -17", -395, 10)

{

Outlier("General", 0.05);

};

C_Date("Coconut Grove/UTh -19", -724, 13)

{

Outlier("General", 0.05);

};

C_Date("Va'oto/UTh -20", -429, 9)

{

Outlier("General", 0.05);

};

C_Date("Va'oto/UTh -21", -429, 9)

{

Outlier("General", 0.05);

};

C_Date("Va'oto/UTh -22", -431, 11)

{

Outlier("General", 0.05);

};

C_Date("Va'oto/UTh -23", -554, 7)

{

Outlier("General", 0.05);

};

C_Date("Va'oto/UTh -24", -417, 10)

{

Outlier("General", 0.05);

};

};

Boundary("End 1");

};

};
